# Oil-in-water nanoemulsion-based ethyl lauroyl arginate nanoparticles exhibit potent antibacterial and antibiofilm activity against oral pathogens

**DOI:** 10.7717/peerj.21174

**Published:** 2026-04-23

**Authors:** Saharut Wongkaewkhiaw, Tanita Pairojana, Sirirat Chansiri, Stephany Tsao, Soontra Panmekiate, Vichittra Vipismakul, Wilairat Worapamorn, Kanokraj Srisukho, Lalita Tangratchatakul, Siriporn Timpawat, Panitan Jumjitvi, Pisut Rimrang, Naiyaphat Nittayasut, Teerapong Yata, Floris J. Bikker

**Affiliations:** 1 School of Dentistry, King Mongkut’s Institute of Technology Ladkrabang, Ladkrabang, Bangkok, Thailand; 2 Premier Innova Co., Ltd., Prawet, Bangkok, Thailand; 3 Department of Oral Biochemistry, Academic Centre for Dentistry Amsterdam (ACTA), University of Amsterdam and Vrije Universiteit Amsterdam, Amsterdam, North Holland, Netherlands

**Keywords:** Ethyl lauroyl arginate, Nanoparticles, Dental biofilm, Oral pathogens

## Abstract

**Background:**

Dental biofilm is a key etiological factor in oral diseases such as dental caries and periodontal disease, and has also been linked to systemic health complications. In this study, ethyl lauroyl arginate nanoparticles (ELANPs) were formulated using an oil-in-water nanoemulsion system to investigate their antibiofilm capacity and cellular cytotoxicity *in vitro*.

**Methods:**

The morphology of ELANPs was examined using transmission electron microscopy (TEM). Particle size, polydispersity index (PDI), and zeta potential were evaluated to assess nanoparticle stability over time. Biofilm matrix reduction was analyzed using confocal laser scanning microscopy (CLSM), and cytotoxicity was assessed using the 3-(4,5-dimethylthiazol-2-yl)-2,5-diphenyltetrazolium bromide (MTT) assay.

**Results:**

ELANPs displayed a spherical morphology with an average diameter of 84.3 ± 2.6 nm. The zeta potential was 46.7 ± 4.6 mV, and the PDI was 0.18 ± 0.01, indicating good colloidal uniformity and stable nanosuspensions. Slight changes in physicochemical properties were observed at days 14 and 30, and overall stability was maintained. ELANPs significantly reduced the biofilm matrix across all tested oral pathogens while maintaining low cytotoxicity toward normal human gingival fibroblasts.

**Conclusion:**

The findings indicate that ELANPs are physicochemically stable, capable of inhibiting oral biofilm formation, and exhibit minimal cytotoxicity, supporting their potential as a safe and effective oral healthcare agent.

## Introduction

Oral biofilms are complex microbial communities that adhere to both oral hard and soft surfaces and are major contributors to dental caries, periodontal disease, and related systemic conditions (Bowen et al., 2018). The biofilm matrix protects pathogenic microorganisms from immune responses and antimicrobial agents, making eradication difficult (Lv et al., 2025). Although agents, such as chlorhexidine (CHX), are available, their limitations, including incomplete biofilm removal and potential cytotoxicity (Wyganowska-Swiatkowska et al., 2016), emphasize the need for new and effective biofilm-control strategies.

Ethyl lauroyl arginate (ELA), also known as ethyl-Nα-lauroyl-L-arginate, is an amino acid–based cationic surfactant synthesized from L-arginine, lauric acid, and ethanol. Its use as a food preservative has been approved by the US Food and Drug Administration (FDA) (Younes et al., 2019). ELA demonstrates antimicrobial effects across diverse microorganisms, including bacteria, yeasts, and filamentous fungi (Ma et al., 2023). ELA primarily targets and disrupts the bacterial cell membrane, resulting in leakage of intracellular contents and subsequent cell death (Becerril et al., 2013). Nonetheless, Gram-negative bacteria typically show greater resistance to ELA than Gram-positive species (Becerril et al., 2013; Rodriguez et al., 2004). Moreover, yeasts and molds have been reported to show even higher levels of resistance compared with bacteria (Loeffler et al., 2014; Ma et al., 2023). These findings highlight the need for strategies that can further enhance the broad-spectrum antimicrobial activity of ELA.

Nanoparticle-based formulations are emerging as a transformative approach in clinical research and therapeutic development (Mondal et al., 2024). In general, nanoparticles are classified as colloidal particles with sizes between 10 nm and <1,000 nm; however, particles below 200 nm are generally preferred for nanomedical use (Biswas et al., 2014). Formulating bioactive agents into nanoparticle systems can enhance their functional properties, including solubility, bioavailability, targeted delivery, physicochemical stability, and the potential to reduce cytotoxicity (Islam et al., 2025). Moreover, their nanoscale dimensions, high surface-area-to-volume ratio, and modifiable surface chemistry (Yang et al., 2019), facilitate improved interactions with microbial cells, thereby enhancing antimicrobial efficacy relative to the native compound (Mondal et al., 2024). Oil-in-water nanoemulsion–based nanoparticle formulations are well established in pharmaceutical applications and are widely regarded as effective drug delivery systems due to their ability to enhance solubility, stability, and bioavailability (Alliod et al., 2018). To the best of our knowledge, the formulation of ELA as nanoparticles using an oil-in-water nanoemulsion system has not previously been reported.

Therefore, this study aimed to formulate ELA into a nanoparticle delivery system (ELA nanoparticles; ELANPs) and to evaluate their physicochemical stability. In addition, the broad-spectrum antimicrobial activity of ELANPs against various oral pathogens in both planktonic and biofilm models, as well as their cytotoxicity toward human gingival fibroblasts, were evaluated *in vitro*. For the first time, ELA was successfully formulated into nanoparticles (ELANPs), which exhibited spherical morphology and nanoscale dimensions. The nanoparticles maintained stability at different temperatures (25 °C and 45 °C) over a 30-day observation period. ELANPs demonstrated broad-spectrum antimicrobial activity against all tested oral pathogens in their planktonic form and effectively eliminated the biofilm matrix. Moreover, ELANPs exhibited low cytotoxicity toward normal human gingival fibroblast cells at concentrations that achieved significant antibiofilm activity.

## Materials & Methods

### Bacterial species and growth conditions

All microorganisms were obtained from the American Type Culture Collection (ATCC^^®^^) (Manassas, VA, USA), including *Streptococcus mutans* ATCC 25175™, *Streptococcus sanguinis* ATCC 10556™, *Streptococcus oralis* ATCC 9811™, *Aggregatibacter actinomycetemcomitans* ATCC 43718™, and *Candida albicans* ATCC 10231™. All bacterial strains were initially cultured on Brain Heart Infusion (BHI) agar (HiMedia^^®^^, Mumbai, India) at 37 °C under 5% CO_2_ for 24 h. A single colony of each bacterial strain was then inoculated into BHI broth and incubated anaerobically at 37 °C in 5% CO_2_ for 18 h (Chamlagain et al., 2024). *C. albicans* was first cultured on yeast extract peptone dextrose (YEPD) agar (1% yeast extract, 2% peptone, and 2% D-glucose) and subsequently maintained in RPMI-1640 medium (HyClone™, Marlborough, MA, USA) supplemented with L-glutamine for biofilm experiments (Zhu et al., 2023).

### Preparation of ethyl lauroyl arginate nanoparticles (ELANPs)

Ethyl lauroyl arginate (five g) was dissolved in purified water (77.5 g) at 60–70 °C under continuous stirring to obtain a clear aqueous phase. Polyethylene glycol (PEG) 4000 (0.5 g) was then added and mixed until fully dissolved. Separately, caprylic/capric triglyceride (10 g), sorbitan monooleate 80 (five g), cetyl alcohol (one g), and cholesterol (one g) were heated at 70–75 °C with stirring until a homogeneous oil phase was formed. The aqueous phase was then combined with the oil phase at the same temperature and mixed thoroughly. The mixture was subsequently subjected to high-shear homogenization followed by ultrasonication to reduce droplet size, resulting in the formation of stable ELANPs.

### Determination of particle size and stability

The physicochemical characteristics and stability of ELANPs were evaluated using dynamic light scattering (DLS), as previously described (Des Bouillons-Gamboa et al., 2024). Particle size, polydispersity index (PDI), and zeta potential were measured to assess nanoparticle dispersion quality and surface charge, which serve as indicators of particle stability (Pochapski et al., 2021). Stability was examined by storing samples at 25 °C (ambient conditions) and 45 °C (accelerated conditions), followed by measurements on day 0, day 14, and day 30. Briefly, 50 µl of ELANPs were diluted in one ml of sterile distilled water and analyzed using a Malvern Panalytical Zetasizer ZSU3200 (Malvern Panalytical, Worcestershire, UK). Measurements were performed under standard conditions (viscosity = 0.89, dielectric constant = 80), and data were processed using ZS Xplorer software (Malvern Panalytical). Three independent experiments were performed, each in triplicate.

### Transmission electron microscope

The morphology of ELANPs was analyzed using the negative staining technique essentially as described before (Wongkaewkhiaw et al., 2022). In brief, a droplet of the ELANP suspension was placed onto a carbon-coated grid and subsequently stained with 1% uranyl acetate for 1 min. The samples were then visualized using a transmission electron microscope (JEM-1400; JEOL, Tokyo, Japan).

### Bacterial susceptibility testing in planktonic culture

The minimum inhibitory concentrations (MICs) and minimum bactericidal concentrations (MBCs) of ELANPs were determined using the broth microdilution method (Wongkaewkhiaw et al., 2019). Two-fold serial dilutions of ELANPs were prepared in BHI broth to achieve final concentrations ranging from five μg/ml to 10,000 μg/ml in 96-well microplates (Nunclon™, Roskilde, Denmark). A 200 µl of inoculum containing 5 × 10^5^ CFU/ml of each overnight culture was added each well and incubated at 37 °C for 24 h. Wells containing microbial cells without ELANPs served as the growth control while a vehicle control was included as a negative control. Following incubation, MIC values were defined as the lowest concentrations showing no visible growth and were confirmed spectrophotometrically at OD_6__2__0_ using a microplate reader (BIOBASE BK-V1200; Biobase, Shandong, China). For MBC determination, aliquots from wells showing no turbidity were plated on BHI agar. Three independent experiments were performed, each in triplicate.

### Quantification of biofilm formation

Biofilm-forming capacity by all tested isolates was evaluated using the crystal violet staining assay as previously described (Anutrakunchai et al., 2022). Briefly, each microorganism was adjusted to a final concentration of 1 × 10^8^ CFU/ml, and 200 µl of the suspension was added to each well of a 96-well plate. Wells containing only the culture medium served as negative controls. The plates were incubated at 37 °C for 3 h to allow initial adhesion. Non-adherent cells were then removed, and each well was replenished with fresh medium followed by an additional 21 h incubation at 37 °C. After 24 and 48 h, biofilms were fixed with 99% methanol for 15 min, stained with 2% crystal violet (Sigma-Aldrich, St. Louis, MO) for 5 min, and rinsed thoroughly with running tap water to remove excess dye. The bound stain was solubilized with 33% (v/v) glacial acetic acid, and the optical density (OD) was measured at 620 nm using a microplate reader (BIOBASE BK-V1200). Each experiment was performed independently three times with eight replicates per condition.

### Biofilm susceptibility testing

The susceptibility of all tested oral pathogens under biofilm-stimulating conditions was evaluated as previously described (Anutrakunchai et al., 2018), with minor modifications. To determine the minimum biofilm inhibitory concentration (MBIC), 200 µl each overnight culture (1 × 10^8^ CFU/ml) were added to sterile 96-well plates (Nunclon™) and covered with a TSP pin lid (NUNC™, Roskilde, Denmark). The plates were incubated at 37 °C for 3 h to allow bacterial attachment, followed by an additional 21 h incubation to establish 24-h biofilms on the pin surfaces. After incubation, the biofilm-coated pins were gently washed with sterile distilled water and transferred to new 96-well plates containing two-fold serial dilutions of ELANPs (5 μg/ml–10,000 μg/ml). The plates were then incubated at 37 °C with 5% CO_2_ for 24 h. Following treatment, the pin lids were removed, and the turbidity of each well was measured at 620 nm using a microplate reader (BIOBASE BK-V1200) to determine the MBIC. To determine the planktonic minimum biofilm inhibitory concentration (pMBIC), aliquots from each well were plated onto BHI agar, and the lowest concentration that yielded no visible bacterial growth was recorded as the pMBIC.

For assessment of the minimum biofilm eradication concentration (MBEC), the residual biofilms attached to the pins were transferred into fresh culture media by sonication for 5 min, after which microbial regrowth was evaluated following 24 h of incubation at 37 °C by measuring turbidity at 620 nm. Three independent experiments were performed, each in triplicate.

### Anti-biofilm formation capacity of ELANPs

Anti-biofilm formation of ELANPs against all tested pathogens was performed as previously described (Bie et al., 2025). A 200 µl of microbial cell suspension (1 × 10^8^ CFU/ml) was incubated with ELANPs at concentrations ranging from 5 μg/ml–10,000 μg/ml at 37 °C under 5% CO_2_ for 24 h. Biofilm biomass was quantified using the crystal violet staining assay, and the percentage reduction was calculated as [1 − (OD_620_ treated/OD_620_ untreated)] × 100%. Three independent experiments were performed, each in triplicate.

### Anti-adherence effect of ELANPs

The effect of ELANPs on microbial attachment was assessed using the static Amsterdam Active Attachment (AAA) model, as previously described (Deng et al., 2009; Lachica et al., 2019). Briefly, 1.5 ml of each overnight culture (10^8^ CFU/ml) was added to a 24-well tissue culture plate (Nunclon™) in the presence of 39 μg/ml ELANPs, which was selected because this concentration reduced biofilm formation by more than 80% in all tested microorganisms. The plate was covered with a sterile stainless-steel lid containing glass coverslips and incubated anaerobically at 37 °C under 5% CO_2_ for 3 h to allow initial attachment. Wells containing only culture medium served as untreated controls. After incubation, the coverslips were gently washed twice with sterile distilled water, and the sessile cells were stained with SYTO 9 (Invitrogen, Carlsbad, CA, USA) to visualize viable cells. Attached cells were subsequently imaged in 2D at the top, middle, and bottom regions of the slide using a confocal laser-scanning microscope (CLSM) (Leica DMi8, Wetzlar, Germany). The ratio of fluorescent intensity between ELANP-treated and untreated samples was quantified using the Leica Application Suite X (LAS X) software. Three independent experiments were performed, each in triplicate.

### Effect of ELANPs on biofilm matrix

The effect of ELANPs on the biofilm matrix was further evaluated using the AAA model, as previously described (Deng et al., 2009). Briefly, 1.5 ml of each overnight culture (10^8^ CFU/ml) was added to a 24-well tissue culture plate (Nunclon™). The plate was covered with a sterile stainless-steel lid containing glass coverslips and incubated anaerobically at 37 °C under 5% CO_2_ for 24 h in the presence of 39 μg/ml ELANPs. A well free of antimicrobial agent was used as a control. After incubation, the biofilm-coated coverslips were gently washed twice with sterile distilled water and fixed with 2.5% glutaraldehyde (Sigma-Aldrich) at 25 °C for 1 h. The fixed biofilms were stained with FITC–ConA (50 μg/ml), which labels the exopolysaccharide matrix, for 30 min (Invitrogen). Biofilm structures were visualized using CLSM (Leica DMi8) at the top, middle, and bottom regions of the slide, and Z-stack images were captured at 0.5 µm intervals throughout the biofilm depth. Biofilm biomass was quantified using COMSTAT analysis (BioCentrum-DTU, Lyngby, Denmark) to evaluate matrix reduction. Three independent experiments were performed, each in triplicate.

### Cytotoxicity testing

Human gingival fibroblast-1 (HGF-1; CRL-2021™, ATCC^^®^^) cells were used to determine the cytotoxicity of ELANPs, as previously described (Bencze et al., 2023). Cells were cultured in Dulbecco’s Modified Eagle’s Medium (DMEM; HyClone™) supplemented with 10% fetal bovine serum (FBS; HyClone™) and maintained at 37 °C in a humidified atmosphere containing 5% CO_2_ for 18 h.

The cells were seeded at a density of 1.0 × 10^4^ cells/well in 96-well plates and grown overnight. Cells were then exposed to 10 μg/ml–5,000 μg/ml ELANPs and 4 μg/ml–2,000 μg/ml (0.0005–0.2%) CHX for 24 h. Cells exposed only to culture medium served as the negative control. Cytotoxicity was evaluated using the MTT assay (0.5 mg/ml) according to the manufacturer’s instructions (Sigma-Aldrich). Absorbance was measured at 570 nm with background subtraction at 630 nm. Three independent experiments were performed, each in triplicate.

### Statistical analysis

Data are presented as mean ± standard deviation (SD). Statistical significance in the stability profile of ELANPs was determined by comparing day 0 with day 14 and day 30 at 25 °C and 45 °C using one-way ANOVA followed by Dunnett’s post hoc test. Comparisons of biofilm-forming capacity, fluorescence intensity, and cellular cytotoxicity between untreated and ELANP-treated groups were analyzed using two-way ANOVA with Sidak’s multiple comparisons test. In addition, statistical differences in biofilm mass reduction were evaluated using an Unpaired *t*-test. All statistical analyses were performed using GraphPad Prism (version 9.0).

## Results

### Characterization and stability of ELANPs

**Figure 1:**
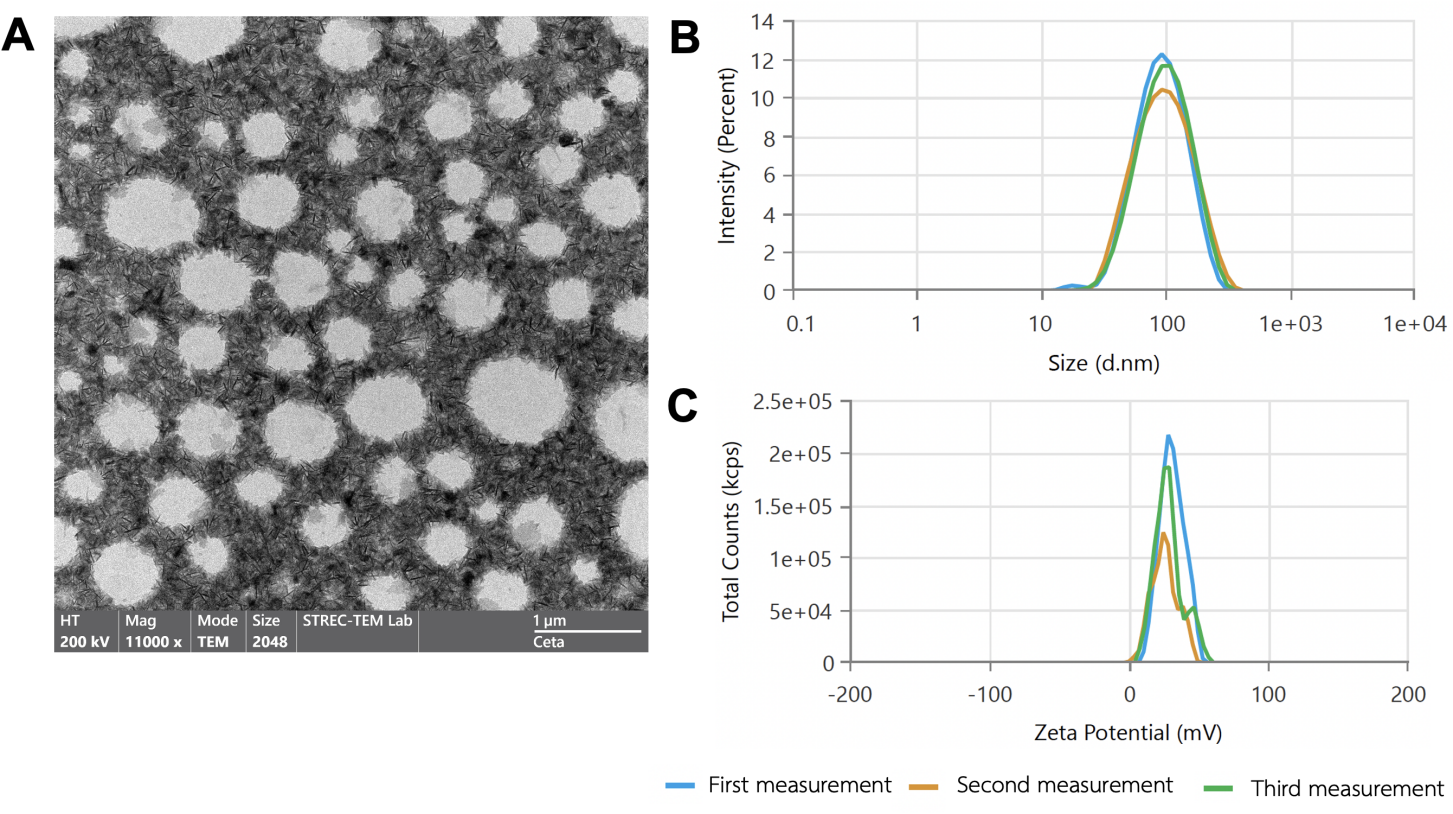
Characterization of ELANPs. (A) Morphology of ELANPs was observed using TEM. Scale bar represents one µm. (B–C) Particle size distribution and zeta potential were evaluated using dynamic light scattering (DLS).

After formulation, the particle size, zeta potential, and PDI were evaluated on day 1. TEM images confirmed that the ELANPs possessed a spherical morphology with predominantly non-aggregated particles (Fig. 1A). The nanoparticles had an average diameter of 84.3 ± 2.6 nm (Table 1), consistent with a uniform nano-sized structure (Fig. 1B). The zeta potential measured 46.7 ± 4.6 mV, and the PDI was 0.18 ± 0.01 (Fig. 1C), indicating good colloidal uniformity and overall nanosuspension stability.

**Table 1:** Physicochemical characteristics and stability profile of ELANPs.

**Sample**	**Temperatures (°C)**	**Particle size** **(Z-average: nm)**	**Zeta potential** **(mV)**	**Polydispersity index (PDI)**
Day 0	–	84.3 ± 2.6	46.7 ± 4.6	0.18 ± 0.01
Day 14	25	89.3 ± 1.5^**^	58.9 ± 2.4^**^	0.16 ± 0.0^ns^
	45	115.5 ± 0.9^***^	34.1 ± 3.4^**^	0.18 ± 0.03^ns^
Day 30	25	110.3 ± 1.2^***^	33.9 ± 4.7^**^	0.14 ± 0.02^ns^
	45	124.1 ± 0.8^***^	28.1 ± 2.6^***^	0.20 ± 0.03^ns^

**Notes.**

Data are presented as mean ± SD of three independent experiments carried out in triplicate. Statistical significance was determined by comparing day 0 with day 14 and day 30 at 25 °C and 45 °C.

ns, not significant.

** *p* < 0.01.

*** *p* < 0.001 (*n* = 3).

### Stability assessment of ELANPs

At day 14, ELANPs stored at 25 °C exhibited minimal physicochemical variation (Table 1), revealed by a slight increase in particle size (89.3 ± 1.5 nm), an elevated zeta potential (58.9 ± 2.4 mV), and a reduced PDI (0.16 ± 0.01), indicating improved size uniformity and sustained colloidal stability. In contrast, under accelerated storage at 45 °C, particle size significantly increased more noticeably (115.5 ± 0.9 nm) (*p* < 0.001) and the zeta potential declined (34.1 ± 3.4 mV) (*p* < 0.01), reflecting reduced electrostatic stability, although the PDI remained within an acceptable range.

By day 30, ELANPs stored at 25 °C remained within the nanoscale range (110.3 ± 1.2 nm) with a low PDI (0.14 ± 0.02) and moderate zeta potential (33.9 ± 4.7 mV), supporting continued stability over the evaluation period. However, samples stored at 45 °C exhibited further increases in particle size (124.1 ± 0.8 nm) (*p* < 0.001) and PDI (0.20 ± 0.03), accompanied by an additional decline in zeta potential (28.1 ± 2.6 mV) (*p* < 0.001), indicating progressive destabilization at elevated temperature. Slight increases in particle size, concomitant reductions in zeta potential, and variations in PDI were observed, potentially resulting from minor aggregation or exposure to thermal stress. Despite these changes, the ELANPs exhibited satisfactory physicochemical stability over a 30-day period.

### MIC and MBC values of ELANPs against planktonic culture

The MIC and MBC values of ELANPs against all tested oral pathogens are shown in Table 2. All isolates in planktonic form were susceptible to ELANPs, as revealed by their MIC values. For most oral pathogens, both MIC and MBC were 20 μg/ml, whereas *A. a* exhibited MIC and MBC values of 10 μg/ml. Moreover, the MBC/MIC ratios for all isolates were <4, confirming the bactericidal activity of ELANPs. No antimicrobial activity was observed in the vehicle (solvent) control. These results demonstrate that ELANPs exhibit broad-spectrum antimicrobial activity and effectively eliminate planktonic oral pathogens.

**Table 2:** Antimicrobial susceptibility of ELANPs against all tested oral pathogens in planktonic and biofilm cultures.

**Microorganisms**	**Planktonic**				**Biofilm**		
	**ELANPs (**μ**g/ml)**		**Vehicle**		**ELANPs (**μ**g/ml)**		
	**MIC**	**MBC**	**MIC**	**MBC**	**MBIC**	**P-MBIC**	**MBEC**
*S. mutans* ATCC 25175	20	20	N/A	N/A	20	39	39
*S. sanguinis* ATCC 10556	20	20	N/A	N/A	20	20	20
*S. oralis* ATCC 9811	20	20	N/A	N/A	39	39	78
*A. a* ATCC 43718	10	10	N/A	N/A	20	78	78
*C. albicans* ATCC 10231	20	20	N/A	N/A	20	78	78

**Notes.**

N/A, Not applicable.

### Biofilm formation

**Figure 2:**
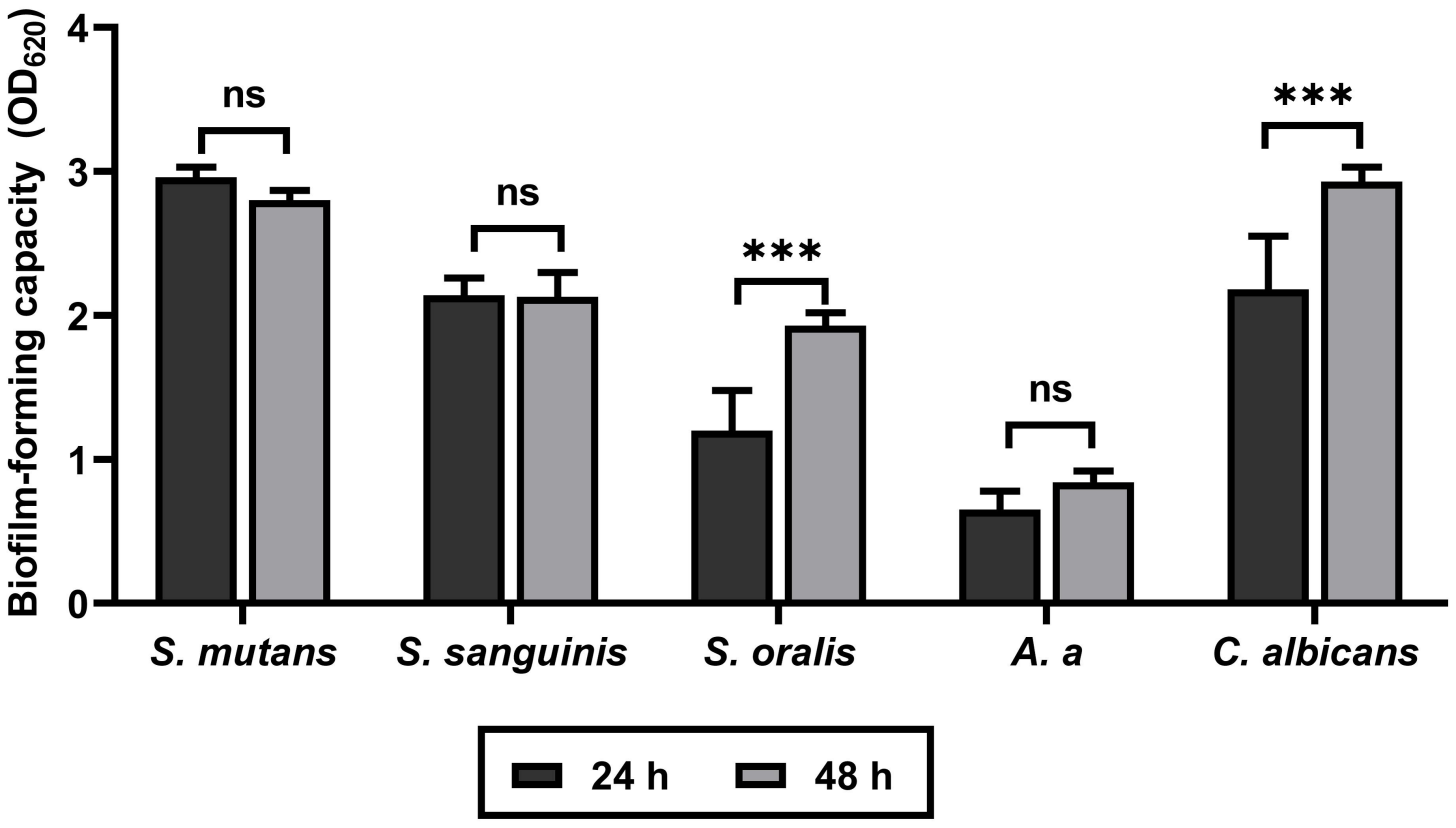
Biofilm-forming capacity of all tested oral pathogens. The 24- and 48-h biofilms were fixed with 99% methanol and stained with 2% crystal violet. Optical density was measured at 620 nm using a microplate reader. Data are presented as means ± SD of three independent experiments carried out in eight replicates. ns = not significant, ***p < 0.001.

The biofilm-forming capacity of all tested isolates at 24 and 48 h is shown in Fig. 2. Crystal violet staining demonstrated a slight reduction in biofilm formation by *S. mutans* at 48 h relative to 24 h. Conversely, time-dependent increases in biofilm formation were observed for *S. oralis*, *A. a*, and *C. albicans*. Only the biofilms produced by *S. oralis* and *C. albicans* showed statistically significant increases (*p* < 0.001). *S. sanguinis* exhibited similar levels of biofilm formation at both time points. These results confirm the biofilm-forming ability of all tested isolates.

### Antimicrobial susceptibility under biofilm-stimulating condition

The biofilm-eradicating effect of ELANPs on 24-h pre-formed biofilms is presented in Table 2. The MBIC values against all tested isolates, ranging from 20–39 μg/ml, indicate effective inhibition of biofilm viability under biofilm-stimulating conditions. Furthermore, at 78 μg/ml, ELANPs eradicated all planktonic cells shed from the biofilms, defined as the pMBIC values, suggesting that ELANPs not only eliminate biofilm-embedded cells but also eradicate planktonic or dispersal cells shed within the biofilm environment. Biofilm eradication by ELANPs was observed at concentrations of 20–78 μg/ml against all tested isolates. These results demonstrate that ELANPs effectively inhibit and eradicate 24-h pre-formed biofilms of all tested isolates.

### Inhibition of biofilm formation

**Figure 3:**
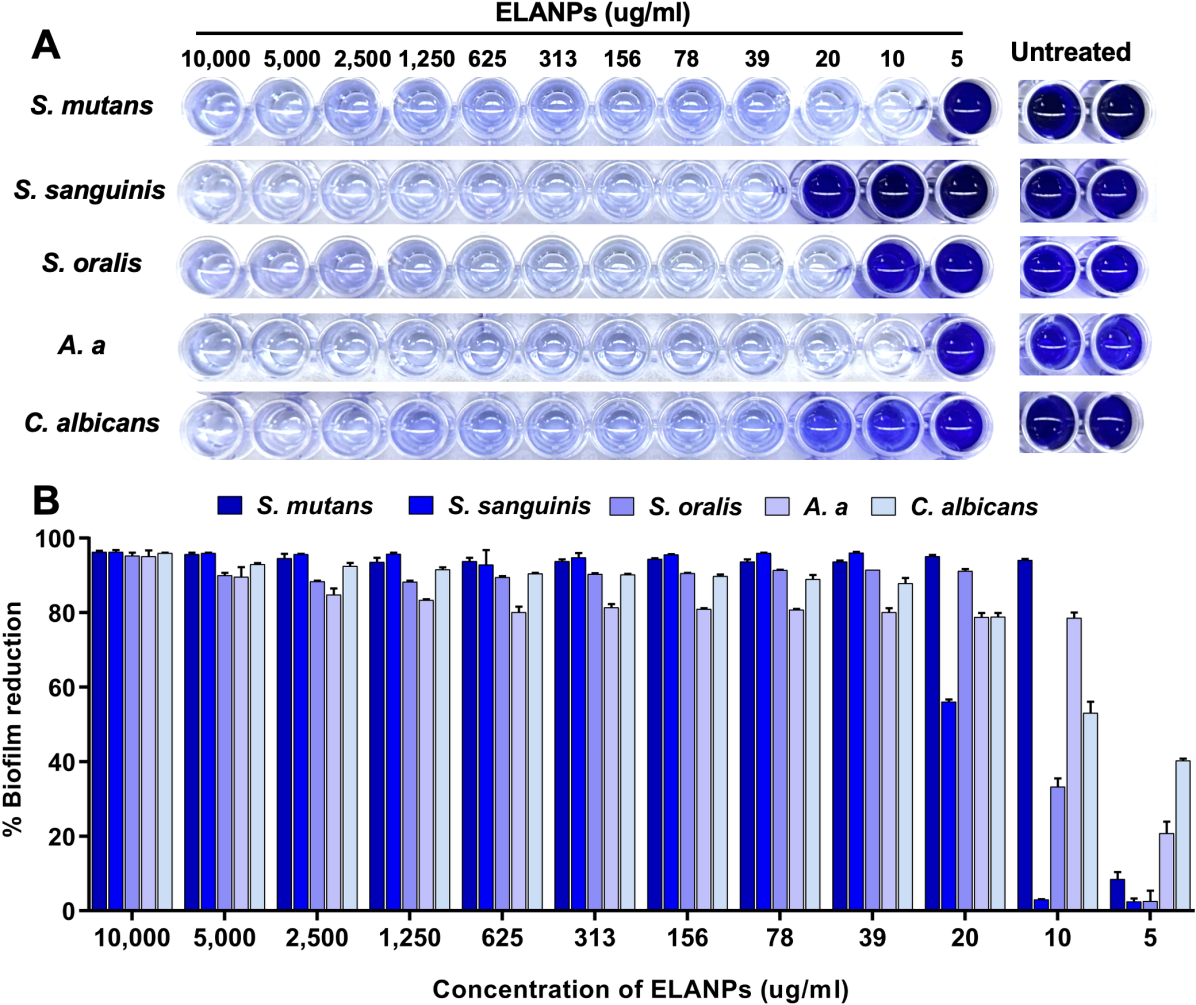
Reduction of biofilm formation. (A) Overnight cultures (1 × 10^8^ CFU/ml) were incubated with ELANPs at concentrations ranging from 5–10,000 μg/ml at 37 ° C under 5% CO_2_ for 24 h. Remaining attached biofilm was quantified using the crystal violet staining assay. (B) Percentage reduction was calculated and presented as means ± SD of three independent experiments carried out in triplicate.

The effects of ELANPs on biofilm reduction were evaluated using the crystal violet staining assay, as shown in Fig. 3. A clear, dose-dependent decrease in biofilm formation was observed for all tested isolates (Fig. 3A). ELANPs demonstrated the highest antibiofilm efficacy against *S. mutans* and *A. a*, followed by *S. oralis*, *C. albicans*, and *S. sanguinis*. At the highest concentration tested (10,000 μg/ml), ELANPs inhibited biofilm formation by more than 95% (Fig. 3B). Notably, even at 10 μg/ml, ELANPs substantially inhibited the biofilms of *S. mutans* (94.1 ± 0.3%) and *A. a* (78.6  ± 1.34%). Moreover, a concentration of 39 μg/ml ELANPs reduced biofilm formation by more than 80% in all microorganisms; therefore, this concentration was selected for subsequent biofilm experiments. Overall, ELANPs exhibited pronounced biofilm-reducing activity, indicating their potent inhibitory effect on biofilm development.

### Interference of microbial attachment

**Figure 4:**
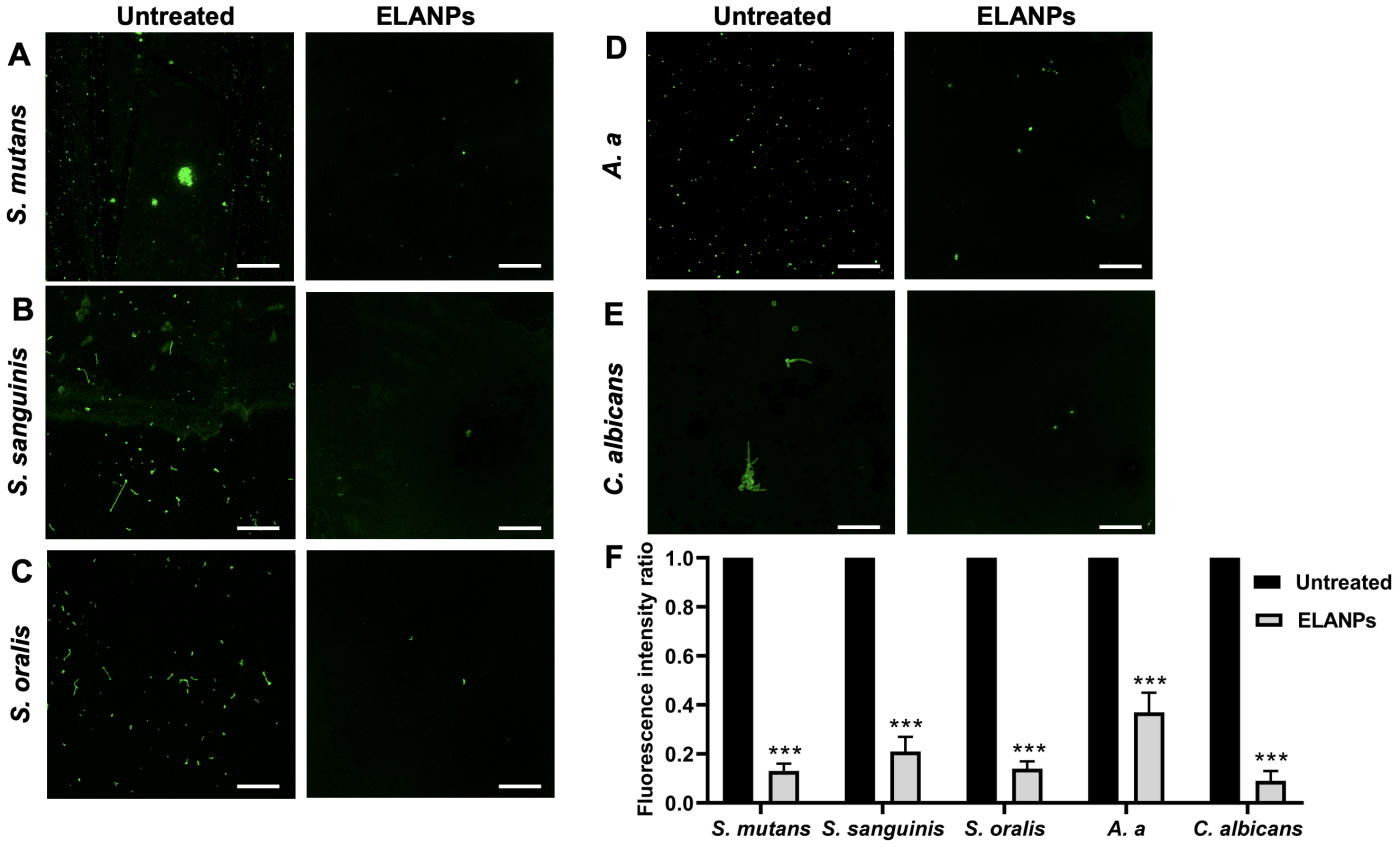
Interference with microbial attachment during early biofilm formation. (A–E) Overnight cultures (1 × 10^8^ CFU/ml) were incubated with ELANPs for 3 h. Sessile cells were stained with SYTO-9 to visualize viable cells and observed as 2D images using CLSM. (F) Fluorescence intensity was quantified using Leica Application Suite X (LAS X) software and expressed as the ratio of ELANP-treated to untreated samples of three independent experiments carried out in triplicate. ***p < 0.001.

The impact of ELANPs on the initial attachment phase of biofilm formation was examined using SYTO-9 staining to visualize viable cells, as shown in Fig. 4. Treatment with ELANPs resulted in reduced viable-cell attachment during early biofilm formation compared with the untreated control across all tested isolates (Figs. 4A–4E). These observations were confirmed by quantitative analysis of the fluorescence intensity ratios between untreated and ELANP-treated groups. A statistically significant decrease in microbial attachment (*p* < 0.001) was observed for all examined oral pathogens (Fig. 4F), demonstrating the strong inhibitory potential of ELANPs against pathogen attachment, which may consequently inhibit subsequent biofilm development.

### Biofilm matrix reduction

**Figure 5:**
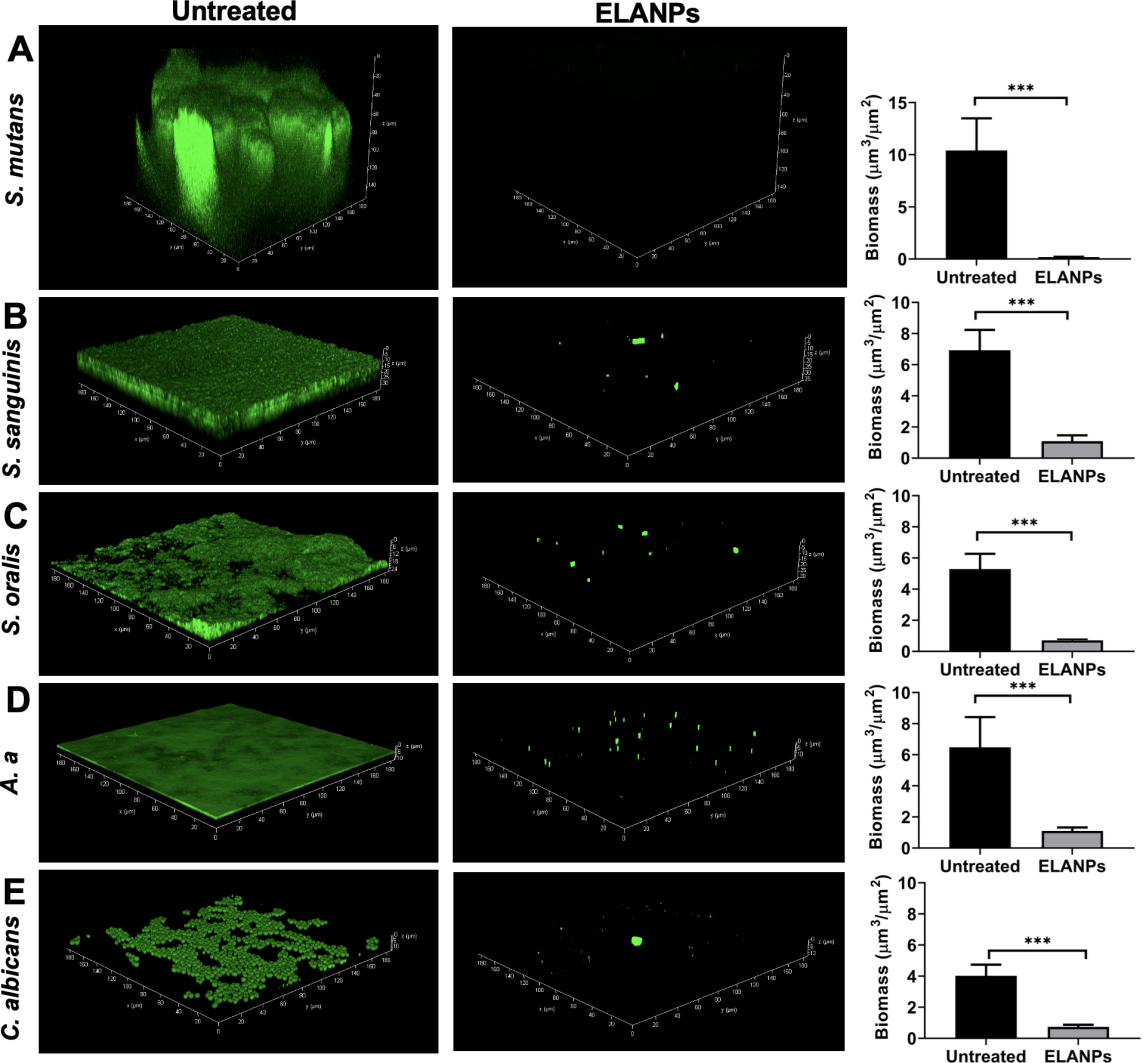
Biofilm disruption by ELANPs. (A–E) Overnight cultures (1 × 10^8^ CFU/ml) were incubated with ELANPs for 24 h. Remaining biofilm matrix was visualized using FITC–ConA, and biomass was quantified using COMSTAT. Data are presented as mean ± SD of three independent experiments carried out in triplicate. ***p < 0.001.

The effect of ELANPs on the biofilm matrix was further evaluated using FITC–ConA staining, with green fluorescence representing the extracellular matrix of each microorganism following ELANP exposure (Fig. 5). Treatment with ELANPs resulted in a distinct reduction in the biofilm matrix of all tested oral pathogens compared with the untreated controls. In the control group, *S. mutans* exhibited a thick, voluminous, and highly complex three-dimensional biofilm structure (Fig. 5A); however, ELANP treatment markedly reduced this architecture. Similarly, the relatively thin, flat biofilms formed by *S. sanguinis* (Fig. 5B), *S. oralis* (Fig. 5C), and *A. a* (Fig. 5D) were substantially reduced in the presence of ELANPs. Furthermore, ELANPs effectively disrupted the dense, biofilm-like cell aggregates characteristic of *C. albicans* (Fig. 5E).

To confirm the biofilm-disrupting effects of ELANPs, biofilm biomass (μm^3^/μm^2^) was quantified. All tested microorganisms showed significant reductions in biomass following ELANP treatment (*p* < 0.001) compared with the controls. These findings highlight the strong biofilm-disruptive activity of ELANPs and emphasize their potential applicability in controlling oral biofilms.

### Cytotoxicity assessment

**Figure 6:**
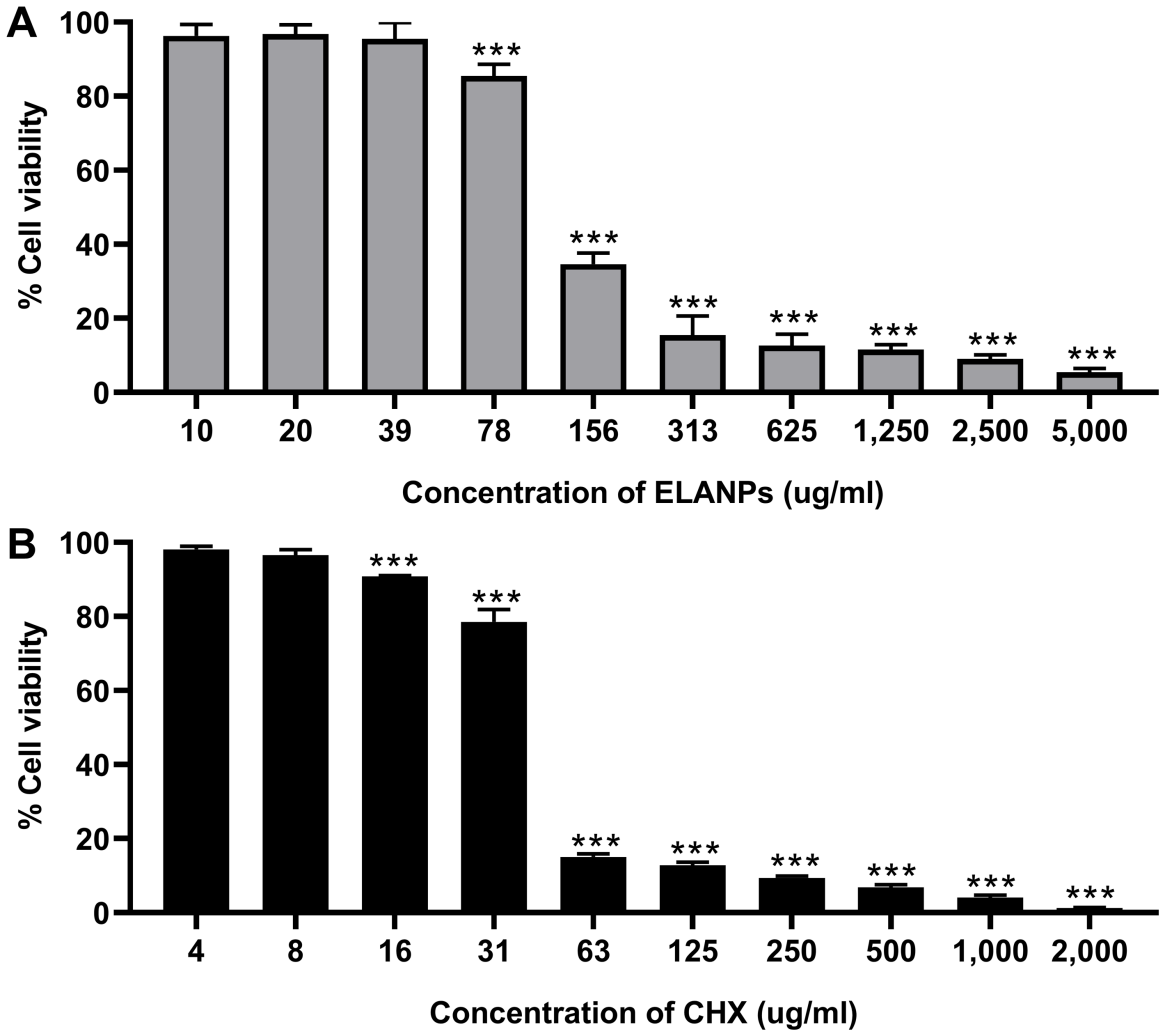
Cytotoxicity of ELANPs and CHX on human gingival fibroblast cells. (A) Cells were treated with ELANPs at concentrations ranging from 10–5,000 μg/ml at 37 °C under 5% CO_2_ for 24 h. (B) CHX was applied at concentrations ranging from 4–2,000 μg/ml (0.0005–0.2%). Cell viability was determined using the MTT assay. Data are presented as mean ± SD of three independent experiments carried out in triplicate. *p < 0.001.

Cell viability was assessed to evaluate the cytotoxic effects of ELANPs and CHX on HGF-1 cells, as shown in Fig. 6. More than 85% of cells remained viable following exposure to 78 μg/ml ELANPs, and over 95% remained viable at 39 μg/ml, indicating that ELANPs possess low cytotoxicity at concentrations effective against oral pathogens in both planktonic and biofilm states. In contrast, treatment with 63–2,000 μg/ml CHX, a commonly used oral antimicrobial agent, resulted in greater than 90% cytotoxicity toward HGF-1 cells, underlining its substantial detrimental effect on host cell viability. Collectively, these findings highlight the markedly superior cytocompatibility of ELANPs and support their potential as a safer alternative to CHX for oral antimicrobial applications.

## Discussion

In the present study, we successfully formulated ELA into ELANPs. We characterized ELANPs and demonstrated that they possess a spherical morphology, uniform colloidal properties, and stable physicochemical characteristics over a 30-day storage period. ELANPs exhibited potent *in vitro* antimicrobial activity against oral pathogens and effectively disrupted biofilm matrix, while maintaining low cytotoxicity toward normal human gingival fibroblast cells.

ELA is recognized as a highly potent antimicrobial compound among emerging food additives, exhibiting broad-spectrum activity against diverse bacteria, yeasts, and filamentous fungi (Ma et al., 2023). Currently, nanoparticle-based formulations are increasingly being explored in clinical applications (Mondal et al., 2024). A recent study formulated ELANPs and demonstrated their ability to reduce total bacterial counts in boar semen extenders (Ngo et al., 2025). Nanoparticle formulations based on oil-in-water nanoemulsions have been shown to improve solubility, stability, and bioavailability, thereby representing a promising class of colloidal drug delivery systems (Alliod et al., 2018; Hwang et al., 2024). However, the nanoparticle characteristics and physicochemical properties of ELANPs formulated using oil-in-water nanoemulsion have not yet been reported. In this study, ELANPs exhibited a spherical morphology with an average particle size of less than 100 nm, indicating a uniform nanoscale structure (Eker et al., 2024). Zeta potential measurements, which provide an estimate of colloidal stability by reflecting the electrostatic repulsive forces between particles, are widely used to assess the tendency of nanoparticles to aggregate (Farooq et al., 2025). Particles with zeta potential values exceeding 40 mV are generally considered to possess good colloidal stability (Viswarupachary et al., 2017). Consistent with this standard, ELANPs in the present study displayed a zeta potential of 46.73 ± 4.63 mV, indicating good to excellent stability. This suggests that ELANPs are unlikely to undergo aggregation or sedimentation, thereby supporting their suitability as a stable colloidal suspension. Another important parameter assessed was the polydispersity index (PDI), which reflects particle size uniformity and indicates whether a formulation is monodisperse. For pharmaceutical and therapeutic applications, a PDI value below 0.3 is generally considered acceptable, as it denotes a homogeneous particle population (Danaei et al., 2018). Consistent with previous recommendations, the PDI of ELANPs was 0.18 ± 0.01, confirming a highly uniform particle size distribution, which is critical for predictable performance, enhanced stability, and consistent drug-delivery behavior.

Particle stability refers to the ability of nanoparticles to maintain their size and surface charge, thereby preventing agglomeration in biological fluids and minimizing undesired cellular interactions (Phan & Haes, 2019). Thus, we further investigated the particle stability on day 14 and day 30. A slight increase in particle size to approximately 80–100 nm was observed at 25 °C on days 14–30, representing an approximate 20% increase. In addition, although the zeta potential decreased (remaining ≥ 30 mV), no significant changes in PDI were detected. In contrast, under accelerated storage at 45 °C, the particle size increased markedly (to approximately 120 nm), and the zeta potential decreased to below 30 mV, indicating reduced colloidal stability. The gradual increase in ELANP size during storage may result from van der Waals forces, Ostwald ripening, and ionic strength–related reductions in colloidal stability common to nanoparticle-based systems (Le-Vinh et al., 2021). The relatively large size of ELANPs may limit their penetration into dense biofilms and their interactions with the salivary pellicle due to hydration layer formation and protein corona effects on organic nanoparticle surfaces (Ikuma, Decho & Lau, 2015). However, previous reports have noted that nanoparticles with sizes up to 150 nm may still retain acceptable colloidal stability (Iyer et al., 2015). These findings indicate that ELANPs maintained acceptable stability despite minor thermal effects, whereas elevated temperatures imposed substantial stress. This highlights the essential challenge of preserving nanoscale dimensions under thermal stress, which often requires optimized excipients, controlled processing, and specialized stabilization strategies. Although physicochemical changes were detected by Day 30, the observed stability supports laboratory-scale preparation and short-term storage, which is consistent with the development of oral healthcare products (Pallavi et al., 2025) and remains relevant for further pharmaceutical optimization of ELANPs.

Previous studies have reported that non-nanoparticle formulations of ELA exhibit antimicrobial activity against various microorganisms (Loeffler et al., 2014) including oral pathogens (Fernández et al., 2018). Although an *in vivo* study of a commercial ELA-containing product demonstrated reduced plaque scores, residual plaque and gingival inflammation persisted after 21 days of use (Valør et al., 2018). These findings suggest that while ELA can partially inhibit bacterial growth, it is insufficient to fully eliminate dental biofilms, consistent with previous observation (Fernández et al., 2018). Given that nanoparticle formulations provide an increased surface area and enhanced interaction with microbial biofilms, thereby improving bactericidal activity (Mondal et al., 2024), we formulated ELA as nanoparticles to enhance its antimicrobial efficacy. In planktonic culture, ELANPs exhibited bactericidal with low MIC/MBC values at 20 μg/ml against all tested pathogens. Although the antimicrobial mechanism of ELA has not been fully elucidated, the bacterial cell membrane is regarded as its primary target (Becerril et al., 2013). The positive charge of its protonated guanidine group enables ELA to interact with anionic membrane proteins (Muriel-Galet et al., 2015; Rodriguez et al., 2004), inducing protein denaturation and increasing membrane permeability. Consequently, intracellular components leak out, ultimately leading to cell death. Several previous studies have shown that Gram-negative bacteria are generally more resistant to ELA than Gram-positive bacteria due to the protective outer membrane, which reduces susceptibility to ELA (Becerril et al., 2013; Rodriguez et al., 2004). Yeasts and molds have also been reported to exhibit substantially higher resistance to ELA compared with bacteria (Loeffler et al., 2014; Ma et al., 2023). In contrast, our findings demonstrate that ELANPs markedly enhanced the broad-spectrum antimicrobial activity of ELA, exhibiting comparable inhibitory effects against Gram-positive and Gram-negative oral pathogens, as well as *C. albicans*, at similar concentrations. These findings indicate that the nanoparticle formulation (ELANPs) substantially enhances the antimicrobial efficacy of ELA, providing a broader spectrum of activity than its native form. Furthermore, the effective elimination of all tested oral pathogens observed in this study suggests that variations in membrane composition do not impede the bactericidal action of ELANPs.

Oral biofilm is a major etiological factor in dental caries and periodontal disease (Bowen et al., 2018); therefore, effective biofilm control is essential for maintaining oral health. Previous studies have demonstrated that ELA exhibits antimicrobial and antibiofilm activity against several foodborne pathogens, including *Listeria monocytogenes* and *Salmonella* spp. (Sadekuzzaman et al., 2017). Consistent with these observations, our findings show that ELANPs markedly reduced the biofilm matrix of all tested oral pathogens, which was further confirmed by biomass quantification. Although the antibiofilm mechanism of ELA has not been fully elucidated, several potential pathways have been proposed. ELA has been reported to inhibit biofilm development in *Pseudomonas aeruginosa* through iron-chelation activity, thereby disrupting iron-dependent signaling essential for biofilm maturation (Kim et al., 2017). In addition, ELA-induced excessive reactive oxygen species (ROS) production has been shown to cause irreversible oxidative damage to DNA, proteins, and lipids in *Escherichia coli* O157:H7 (Yang et al., 2019). However, membrane disruption is still recognized as a key antibacterial mechanism, enabling the elimination of biofilm-embedded cells, including slow-growing or dormant subpopulations that typically exhibit tolerance to conventional antibiotics (Becerril et al., 2013). In this study, ELANPs demonstrated strong antibiofilm activity, with 39 μg/ml identified as the MBIC and 78 μg/ml as the MBEC, effectively inhibiting biofilm development and eliminating 24-h pre-formed biofilms. Taken together, these mechanisms likely contribute to the strong antibiofilm activity of ELANPs observed in this study, including disruption of the biofilm matrix, killing of embedded oral pathogens, and interference with intracellular signaling that may further suppress biofilm development. However, antimicrobial mechanisms may differ between free compounds and their nanoparticle formulations due to unique physicochemical properties (*e.g.*, nanoscale size, high surface-area-to-volume ratio, and modified surface chemistry) (Yang et al., 2019). Thus, the precise mechanisms underlying the antibiofilm effects of ELANPs warrant further investigation. Additionally, future studies with multispecies or saliva-derived biofilms are needed to assess the translational potential of ELANPs.

Reduction of dental plaque formation alone is insufficient for defining an ideal mouthwash or oral healthcare agent; its cytotoxicity must also be carefully evaluated (Çoğulu et al., 2025). Although several biological properties of ELA have been reported, data regarding its cellular cytotoxicity remain limited. In the present study, the nanoparticle formulation of ELA exhibited low cytotoxicity toward normal human gingival fibroblasts after 24 h of exposure, whereas the conventional oral antiseptic CHX demonstrated substantial cytotoxic effects, consistent with previous reports (Dinu et al., 2024; Wyganowska-Swiatkowska et al., 2016). Similar findings in other studies have shown that nanoparticle formulations often exhibit lower cytotoxicity than their corresponding free compounds (Antonio et al., 2017). Taken together, these findings indicate that ELANPs represent a safe and promising alternative antimicrobial agent, supporting their potential for further development as an effective and biocompatible oral healthcare intervention.

## Conclusions

In conclusion, this study demonstrated that ELANPs show good nanoparticle characteristics, including uniform morphology and stable physicochemical properties, indicative of robust formulation stability. ELANPs exhibited strong antimicrobial activity against planktonic cells and effectively eradicated the biofilm matrix formed by all tested oral pathogens, while maintaining low cytotoxicity toward normal human gingival fibroblasts. Collectively, these findings suggest that ELANPs represent a promising candidate for further development as a safe and effective oral healthcare agent.

## Supplemental Information

10.7717/peerj.21174/supp-1 Supplemental Information 1 Biofilm formation

10.7717/peerj.21174/supp-2 Supplemental Information 2 Reduction of biofilm formation

10.7717/peerj.21174/supp-3 Supplemental Information 3 Fluorescence intensity

10.7717/peerj.21174/supp-4 Supplemental Information 4 Biomass

10.7717/peerj.21174/supp-5 Supplemental Information 5 Cytotoxicity

## Additional Information and Declarations

### Data Availability

The following information was supplied regarding data availability: The raw measurements are available in the Supplemental Files.
